# Cordycepin prevents oxidative stress-induced inhibition of osteogenesis

**DOI:** 10.18632/oncotarget.6072

**Published:** 2015-10-10

**Authors:** Feng Wang, Peipei Yin, Ye Lu, Zubin Zhou, Chaolai Jiang, Yingjie Liu, Xiaowei Yu

**Affiliations:** ^1^ Department of Orthopaedic Surgery, Shanghai Jiao Tong University Affiliated Sixth People's Hospital, Shanghai, China

**Keywords:** cordycepin, osteoprotection, oxidative stress, Wnt pathway, Pathology Section

## Abstract

Oxidative stress is known to be involved in impairment of osteogenesis and age-related osteoporosis. Cordycepin is one of the major bioactive components of *Cordyceps militaris* that has been shown to exert antioxidant and anti-inflammatory activities. However, there are few reports available regarding the effects of cordycepin on osteogenesis and the underlying mechanism. In this study, we investigated the potential osteoprotective effects of cordycepin and its mechanism systematically using both *in vitro* model as well as *in vivo* mouse models. We discovered that hydrogen peroxide (H_2_O_2_) induced inhibition of osteogenesis which was rescued by cordycepin treatment in human bone marrow mesenchymal stem cells (BM-MSCs). Cordycepin exerted its protective effects partially by increasing or decreasing expression of osteogenic and osteoclastogenesis marker genes. Treatment with cordycepin increased Wnt-related genes' expression whereas supplementation of Wnt pathway inhibitor reversed its protective effects. In addition, administration of cordycepin promoted osteogenic differentiation of BM-MSCs by reducing oxidative stress in both ovariectomized and aged animal models. Taken together, these results support the protective effects of cordycepin on oxidative stress induced inhibition of osteogenesis by activation of Wnt pathway.

## INTRODUCTION

Oxidative stress is described as an imbalance between excess production of ROS and free radicals (FR) and insufficient antioxidant system function [1]. Several pieces of evidence have suggested a link between oxidative stress, bone formation and osteogenic differentiation [2]. It has been reported that oxidative stress impairs bone mineral density in aged human subjects and reduces osteogenic differentiation of murine pre-osteoblastic and bone marrow-derived stromal cell lines which can be restored by antioxidants [3-5], suggesting that oxidative stress plays an important role in bone injury and age-related osteoporosis. It is well documented that ROS regulates various signaling pathways involved in osteogenesis, including Wnt/β-catenin, MAPK and Hedgehog pathways [2].

It is now known that human bone marrow (BM) harbors a rare population of mesenchymal stem cells (MSC) which retains ability to self-renew and to differentiate into multiple tissues [6]. MSCs exhibit immunomodulatory properties, thereby emerging as attractive candidates for various therapeutic applications [7], especially regenerative medicine. It has been shown that allogeneic bone marrow transplantation in children with defective osteogenesis allowed engraftment of functional donor MSCs, resulting in increased bone marrow density [8]. Therefore, BM-MSCs are believed to hold great therapeutic potential in fracture injury and bone regeneration [9].

Cordycepin, a derivative of the nucleoside adenosine, is one of the major bioactive components of *Cordyceps militaris* [10]. Cordycepin has been shown to have a variety of biological functions, including anti-tumor, antiviral, anti-oxidant, and anti-inflammatory activities [11-14]. Importantly, cordycepin has been shown to attenuate age-related oxidative stress and enhances antioxidant capacity in rats [10]. It has also been shown to prevent rat hearts from ischemia/reperfusion injury partially by activating antioxidant defense response via upregulation of heme oxygenase (HO-1) expression [15]. However, there are few reports available regarding the effects of cordycepin on osteogenesis and its potential mechanism.

Wnt pathway acts as a fundamental signaling pathway that regulates cell proliferation, cell polarity and cell fate determination during embryonic development and tissue homeostasis [16]. Dysregulation of this pathway has been linked to congenital malformation, cancer, osteoporosis and other diseases [17]. Recently, accumulating evidence showed that activation of canonical Wnt signaling led to enhanced bone density whereas disruption of its activation impaired osteogenesis primarily through regulating its downstream target genes, such as β-catenin, cyclin D1, etc [18, 19].

Ovariectomized (OVX) mice are female mice whose ovaries have been removed. OVX mouse model has been used as a typical experimental model for investigation of postmenopausal osteoporosis due to estrogen deficiency in women. In our study, we investigated the potential osteoprotective effects of cordycepin and the underlying mechanism systematically using BM-MSCs as *in vitro* model and OVX as well as aged mouse model as *in vivo* models.

## RESULTS

### Effects of H_2_O_2_ and cordycepin treatment on cell viability of human BM-MSC

**Figure 1:**
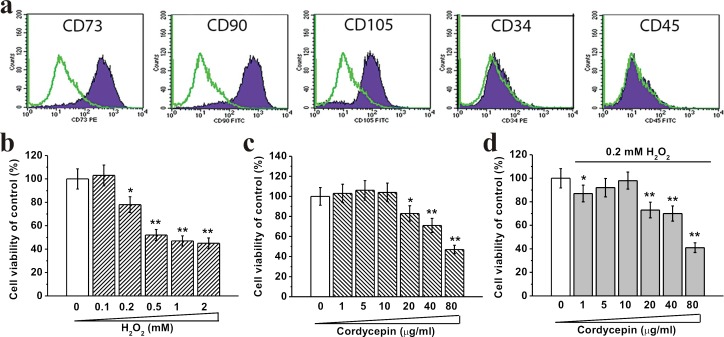
Human BM-MSC viability under different concentrations of H_2_O_2_ and cordycepin treatment at DIV 5 **a.** Characterization of BM-MSCs by flow cytometry. The majority of the cells are CD73^+^, CD90^+^, CD105^+^, CD34^−^ and CD45^−^, which are typical characteristic phenotypes of BM-MSCs. Effects of different concentrations of H_2_O_2_ (0.1, 0.2, 0.5, 1 and 2 mM) exposure for the first 24 hours during the culture. **b.**, different concentrations of cordycepin (1, 5, 10, 20, 40 and 80 μg/mL) without 0.2 mM H_2_O_2_ exposure **c.** and with 0.2 mM H_2_O_2_ exposure **d.** on BM-MSC viability, measured by MTT assay. Data were presented as mean ± S.E.M. *p < 0.05 and **p < 0.01 *versus* control.

We first characterized BM-MSCs by flow cytometry and observed that majority of the cells were CD73^+^, CD90^+^, CD105^+^, CD34^−^ and CD45^−^, which are typical characteristic phenotypes of BM-MSCs (Figure 1a). Oxidative damage was induced by treating BM-MSCs with increasing concentrations of H_2_O_2_ (0.1, 0.2, 0.5, 1 and 2 mM) for 24 hours. 0.2 mM and higher concentrations of H_2_O_2_ significantly decreased cell viability in these cells (Figure 1b). Treatment with cordycepin alone at concentration of 10 μg/mL or lower did not affect cell viability (Figure 1c). Importantly, 5 μg/mL or 10 μg/mL of cordycepin in cells with 0.2 mM H_2_O_2_ exposure increased cell viability compared to cells without cordycepin treatment, indicating that cordycepin can partially inhibit H_2_O_2_ induced cytotoxicity (Figure 1d). Also, the BM-MSCs presented healthy growth and the population doubling time was ∼64 hours (Figure S1).

### Cordycepin protects against H_2_O_2_ induced inhibition of osteogenic differentiation of human BM-MSC *in vitro*

**Figure 2:**
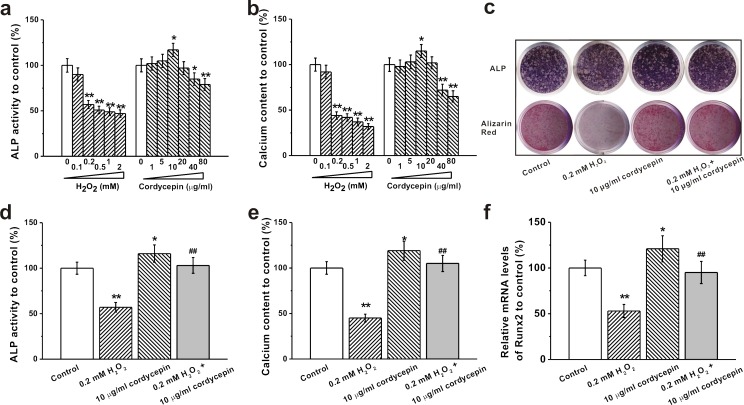
Effects of different concentrations of H_2_O_2_ (0.1, 0.2, 0.5, 1 and 2 mM) and cordycepin (1, 5, 10, 20, 40 and 80 μg/mL) on osteogenic differentiation of human BM-MSCs The osteogenic differentiation was characterized by ALP activity **a.** at DIV 8 and calcium content **b.** at DIV 16. **c.** Representative images for ALP staining in the cultures at DIV 8 and Alizarin red staining in the cultures at day 16 in control, 0.2 mM H_2_O_2_ treated group, 10 μg/mL cordycepin treated group and 0.2 mM H_2_O_2_ +10 μg/mL cordycepin co-treated group. The relative ALP activity **d.**, calcium content **e.** and mRNA levels of Runx2 **f.** to control in these four groups in (c). Data were shown as mean ± S.E.M. *p < 0.05 and **p < 0.01 *versus* control, ##*p* < 0.01 *versus* 0.2 mM H_2_O_2_ treated group.

We investigated protective effects of cordycepin against H_2_O_2_ induced inhibition of osteogenic differentiation by measuring ALP activity and calcium content. 0.2 mM and higher concentrations of H_2_O_2_ or 40 μg/mL and higher concentration of cordycepin alone significantly decreased ALP activity and calcium content whereas 10 μg/mL of cordycepin increased ALP activity and calcium content (Figure 2a and 2b). Co-treatment of 10 μg/mL of cordycepin reversed H_2_O_2_-induced dysfunction as demonstrated by increased ALP staining, activity and calcium content (Figure 2c-2e). ALP activity and calcium content, which are indicators for early and late osteogenesis, are measured 8 and 16 days after cell culture, respectively. Meanwhile, as an essential transcription factor for osteogenesis, runt-related transcription factor 2 (Runx2) was examined as well. The mRNA levels of Runx2 were inhibited in the presence of 0.2 mM H_2_O_2_, while co-treatment with 10 μg/mL of cordycepin successfully restored the mRNA levels of Runx2 (Figure 2f).

### Effects of cordycepin on the expression of osteogenic markers

**Figure 3:**
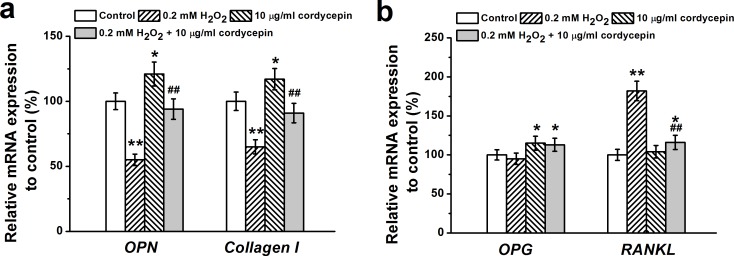
Osteogenic differentiation of human BM-MSCs treated by 0.2 mM H_2_O_2_, 10 μg/mL cordycepin and 0.2 mM H_2_O_2_ +10 μg/mL cordycepin co-treatment Relative *OPN*, *Collagen I* mRNA expression **a.** and *OPG* and *RANKL* mRNA expression in the experimental groups were characterized by RT-PCR analysis. Gene expression was normalized to *GAPDH*. Data were shown as mean ± S.E.M. *p < 0.05 and **p < 0.01 *versus* control, ##*p* < 0.01 *versus* 0.2 mM H_2_O_2_ treated group.

Based on the dose-related protective effects of cordycepin above, 10 μg/mL cordycepin was chosen for the following studies. We further evaluated the effects of 10 μg/mL cordycepin on the expression of osteogenic marker genes using RT-PCR. The results showed that the expression of two osteogenic genes, *OPN* and *Collagen I*, were significantly upregulated in cordycepin treated cells compared to cells treated with 0.2 mM of H_2_O_2_ alone (Figure 3a). Additionally, cordycepin markedly increased the expression of *OPG* but reduced expression of *RANKL* compared to H_2_O_2_ alone treated cells (Figure 3b). *OPG* inhibits osteoclast differentiation by binding to *RANKL*. The ratio between mRNA expressions of *OPG* to *RANKL* is usually used as an indicator of osteoclastogenesis inhibition [20]. Therefore, the above results suggest that 10 μg/mL cordycepin enhances osteogenic differentiation partially through promoting expression of osteogenic markers and inhibition of osteoclastogenesis.

### Cordycepin exerts anti-oxidant effects

**Figure 4:**
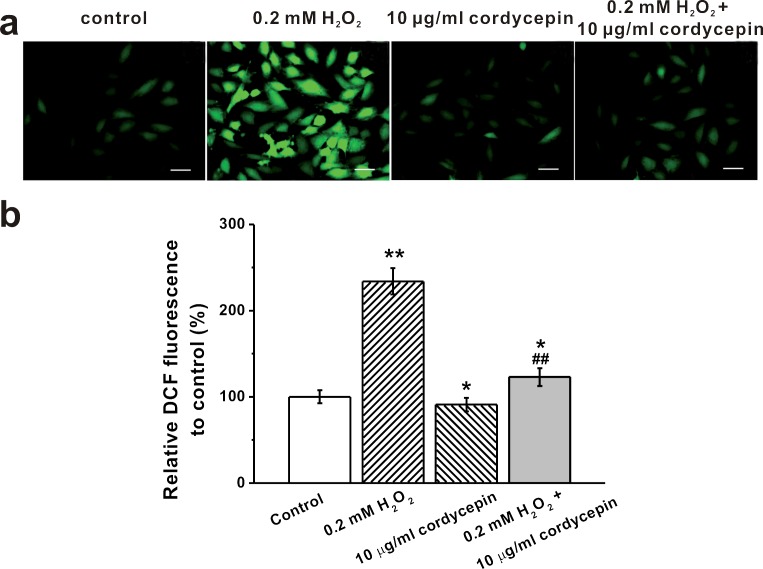
Intracellular ROS levels of human BM-MSCs under 0.2 mM H_2_O_2_ and 10 μg/mL cordycepin exposure, determined by DCF fluorescence **a.** Representative photographs of DCF fluorescence in the experimental groups. Scale bar = 10 μm. **b.** Relative DCF fluorescence was quantitatively analyzed and expressed as percent units of DCF fluorescence of the control. Data were shown as mean ± S.E.M. *p < 0.05 and **p < 0.01 *versus* control, ##*p* < 0.01 *versus* 0.2 mM H_2_O_2_ treated group.

We next examined whether cordycepin exerted anti-oxidant effects using DCF fluorescence quantification assay. As shown in the representative photographs of DCF fluorescence as well as the relative DCF quantification, 10 μg/mL cordycepin markedly reduced intracellular ROS levels induced by H_2_O_2_ treatment (Figure 4). Treatment with cordycepin alone did not exhibit dramatic effects on ROS level (Figure 4b).

### Involvement of Wnt pathway in the osteoprotective effects of cordycepin on human BM-MSCs

**Figure 5:**
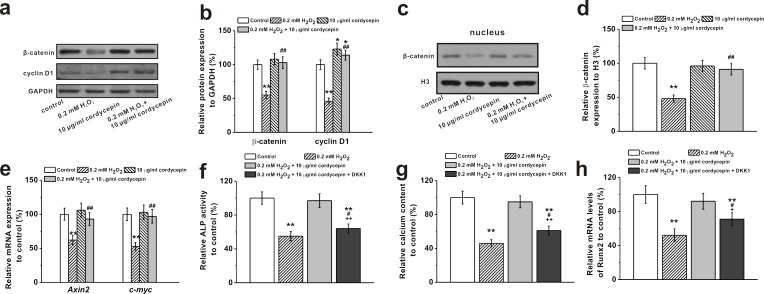
Wnt pathway was involved in the protective effects of cordycepin on the inhibition of osteogenic differentiation of human BM-MSCs induced by 0.2 mM H_2_O_2_ Western blot analysis **a.** and the relative protein expression **b.** of Wnt pathway-related regulators β-catenin and cyclin D1 in control, 0.2 mM H_2_O_2_ treated group, 10 μg/mL cordycepin treated group, and 0.2 mM H_2_O_2_ + 10 μg/mL cordycepin co-treated group. **c.**, **d.** Representative western blot bands of β-catenin and the relative expression in the nucleus. **e.** Relative mRNA expression of Axin2 and c-myc in the experimental groups. Wnt pathway inhibitor, DKK-1 (0.2 μg/ml), could greatly depressed the effects of cordycepin on the inhibition of osteogenic differentiation by H_2_O_2_ exposure, as evidenced by ALP activity **f.,** calcium contents **g.** and Runx2 mRNA expression **h.** Data were shown as mean ± S.E.M. *p < 0.05 and **p < 0.01 *versus* control, #*p* < 0.05 and ##*p* < 0.01 *versus* 0.2 mM H_2_O_2_ treated group, +*p* < 0.05 and ++*p* < 0.01 *versus* 0.2 mM H_2_O_2_ + 10 μg/ml cordycepin co-treated group.

It has been reported that ROS regulates signaling cascades implicated in osteogenesis, such as Wnt pathway [2]. Western Blot analysis showed that 0.2 mM of H_2_O_2_ treatment reduced protein level of Wnt pathway related regulators β-catenin and cyclin D1, which was rescued by 10 μg/mL cordycepin co-treatment (Figure 5a and 5b). Meanwhile, cordycepin successfully reversed the H_2_O_2_-induced inhibition of expression of β-catenin in the nucleus (Figure 5c and 5d), implicating the involvement of Wnt pathway in the protective effects of cordycepin. This was further verified by the results from two other gene markers of Wnt signaling pathway, Axin2 and c-myc. H_2_O_2_ treatment depressed the mRNA levels of both Axin2 and c-myc, while cordycepin co-treatment restored their expressions to control level (Figure 5e). Additionally, supplementation of Wnt pathway inhibitor, DKK1 (0.2 μg/mL), reversed the protective effects of cordycepin as demonstrated by the decreased ALP activity, calcium content and Runx2 mRNA levels compared to cordycepin treated cells (Figure 5f, 5g and 5h), further confirming the possible involvement of Wnt pathway.

### Cordycepin attenuates oxidative stress *in vivo*

**Figure 6:**
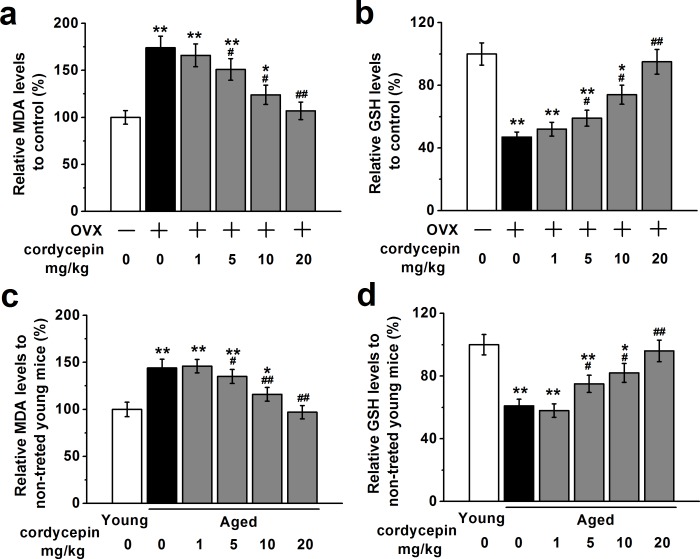
Cordycepin attenuates oxidative stress in ovariectomized **a.**, **b.** and aged animal models **c.**, **d.** (*n* = 8 in all groups), measured by relative serum MDA levels and GSH levels. Data were shown as mean ± S.E.M. *p < 0.05 and **p < 0.01 *versus* control or non-treated young mice, #*p* < 0.05 and ##*p* < 0.01 *versus* OVX group or non-treated aged mice.

Next we investigated effects of cordycepin on oxidative stress *in vivo* using OVX mouse model and aged mouse model by measuring relative serum MDA levels and GSH levels. OVX or aged mice were injected with either vehicle or increasing concentration of cordycepin (1, 5, 10 and 20 mg/kg). OVX or aged receiving vehicle showed significantly higher MDA levels and lower GSH levels compared with control or young non-treated mice whereas cordycepin administration reversed these effects in a dose-dependent manner (Figure 6). These results suggest that cordycepin can attenuate oxidative stress *in vivo*.

### Effects of cordycepin on *in vivo* osteogenic differentiation of human BM-MSCs

**Figure 7:**
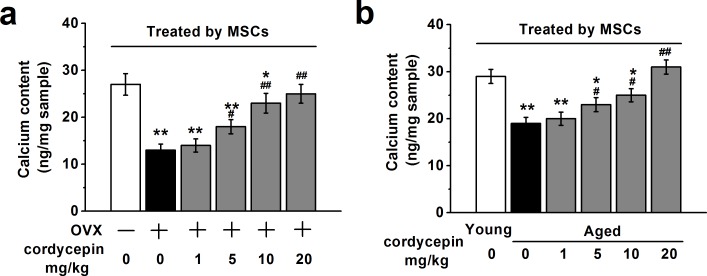
Effects of cordycepin (1, 5, 10 and 20 mg/kg) on *in vivo* osteogenic differentiation of human BM-MSCs in ovariectomized **a.** and aged animal models **b.** The calcium contents in the implantation specimens were determined by spectrophotometry using Methylxylene blue method in the experimental groups (all *n* = 8). Data were shown as mean ± S.E.M. *p < 0.05 and **p < 0.01 *versus* control or non-treated young mice, #*p* < 0.05 and ##*p* < 0.01 *versus* OVX group or non-treated aged mice.

To evaluate the effects of cordycepin on *in vivo* osteogenic differentiation of MSCs, we measured the calcium content in the implantation specimens from OVX and aged mice described above injected with vehicle or increasing concentration of cordycepin (1, 5, 10 and 20 mg/kg). As shown in Figure 7, specimens derived from OVX or aged mice showed decreased calcium content compared to control or young non-treated mice. Treatment with increased concentration of cordycepin increased calcium content in a dose dependent manner, suggesting that cordycepin can promote osteogenesis *in vivo*.

### Cordycepin augments *in vivo* bone formation induced by BM-MSCs

**Figure 8:**
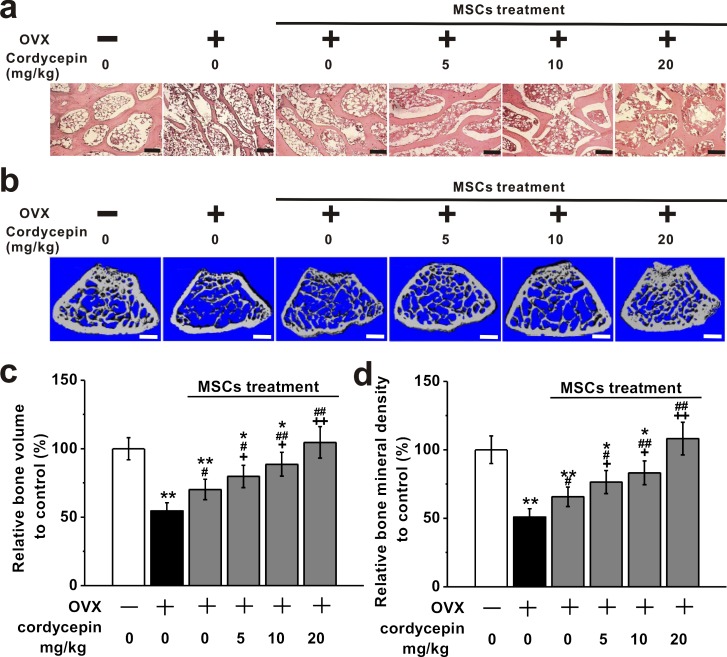
Effects of cordycepin (5, 10 and 20 mg/kg) on bone loss in ovariectomized animal model when treated with MSCs The animals were examined at 8 weeks after ovariectomy. **a.** H&E staining of the femoral trabecular micro architecture in the experimental groups. Scale bar, 100 μm. **b.** Representative images of reconstructed micro-computed tomography of the femur in the experimental groups. Scale bar, 400 μm. Analysis of relative bone volume **c.** and bone mineral density **d.** in the experimental animals. Data were shown as mean ± S.E.M. *p < 0.05 and **p < 0.01 *versus* control mice, #*p* < 0.05 and ##*p* < 0.01 *versus* OVX mice, +*p* < 0.05 and ++*p* < 0.01 *versus* OVX and MSC treated mice.

To further study the effects of cordycepin on *in vivo* bone formation induced by BM-MSCs in OVX animal model, H&E staining and micro-computed tomography (micro-CT) examination were employed in OVX-treated mice. As shown in the H&E staining images (Figure 8a), the control mice showed normal compactness of the diaphysis and competent trabeculae, while OVX mice showed sparse, uniform thinning of trabeculae. The disappearance and loss of connectivity resulted in bigger intertrabecular spaces. This was obviously improved by MSC treatment, especially when co-treated with cordycepin (5, 10 and 20 mg/kg). Micro-CT examination was used to detect the bone mass and bone mineral density in the experimental groups (Figure 8b). As shown in Figure 8c and d, OVX treatment (*n* = 8) induced a significant loss in bone volume and bone mineral density compared to control (*n* = 8). MSC treatment (*n* = 8) significantly increased the bone volume and bone mineral density, while co-treatment with cordycepin at 5, 10 or 20 mg/kg further greatly improved bone volume and bone mineral density in a dose-dependent manner. These results indicate that cordycepin is able to augment *in vivo* bone formation induced by MSCs.

## DISCUSSION

Many reports have provided evidence that there is a link between oxidative stress, bone formation and osteogenic differentiation. It has been shown that oxidative stress impairs osteogenic differentiation of murine pre-osteoblastic and bone marrow-derived stromal cell lines [3]. Exogenous addition of H_2_O_2_ (125-500 μM) to human BM-MSCs reduced Alp activity, a marker for osteogenic differentiation and abolishes osteogenesis in osteoblast progenitors [21, 22]. Additionally, NOX4 (NADPH oxidase 4) knockout mice exhibit higher bone density. NOX4 promotes the transformation of osteoblasts to osteoclasts, thus limiting bone mass [23]. Consistent with these findings, we observed that exposure to H_2_O_2_ (0.2 mM or higher concentration) increased intracellular ROS levels and reduced ALP activity, calcium content as well as Runx2 mRNA levels, suggesting that oxidative stress inhibits osteogenesis in these BM-MSCs.

It has been reported that cordycepin can protect against estrogen deficiency-induced osteoporosis in OVX rats [24]. Another study showed that cordycepin can prevent hearts from ischemia/reperfusion injury via activation of Akt/GSK-3b/p70S6K pathways and HO-1 expression, an antioxidant enzyme, in rat hearts [15]. Additionally, cordycepin has been reported to exert neuroprotective effects by inhibiting glutamate-induced oxidative apoptosis in HT22 cells [14]. Our results showed that co-treatment with cordycepin significantly promoted osteogenesis by reducing production of ROS induced by H_2_O_2_ exposure in BM-MSCs. However, treatment with cordycepin alone did not have effects, demonstrating that cordycepin can exert osteoprotective effects in the presence of excess oxidative stress.

Osteoblasts and osteoclasts are responsible for bone formation and bone resorption, respectively, the two critical processes involved in bone remodeling. *OPG* inhibits osteoclast differentiation by binding to *RANKL* (receptor activator of nuclear factor-B ligand), which is critical for the maturation and activity of osteoclasts [25]. Several studies have shown that ROS can increase expression of *RANKL* in osteoblasts [21]. An increase in *OPG;RANKL* ratio favors bone formation whereas a decrease in this ratio favors bone resorption [20]. We observed that cordycepin significantly increased *OPN* expression but reversed the H_2_O_2_ induced *RANKL* upregulation to control level. Moreover, cordycepin upregulated the expression of *OPG* and *Collagen I,* two osteogenesis marker genes. These results indicate that cordycepin can inhibit osteoclastogenesis and promote osteogenic differentiation by increasing or decreasing related genes' expression.

Current studies support that Wnt signaling pathway plays important roles in osteogenic differentiation and bone formation [26, 27]. Activation of canonical Wnt signaling results in higher bone density whereas disruption of its activation impairs osteogenesis [18, 19]. Wnt signaling pathway contributes to osteogenesis primarily through activation of β-catenin, which induces essential signals for osteogenic initiation and reduces the expression of C/EBPa and PPARg, key suppressors of osteogenesis [2]. In this study, treatment with cordycepin increased protein expression of β-catenin and cyclin D1, another key target gene of Wnt pathway [28]. Additionally, supplementation of DDK1, a Wnt pathway inhibitor, reversed osteoprotective effects of cordycepin. These results suggest that Wnt- β-catenin signaling pathway may be involved in cordycepin promoted osteogenesis of BM-MSCs.

Growing evidence supports that oxidative stress is one of the main contributing factors to decline in cellular functions due to aging [29]. The increased oxidative stress has been associated with the pathogenesis of age-related bone loss in humans and mice [30]. Importantly, cordycepin has recently been shown to mitigate age-related oxidative stress by enhancing antioxidant capacity in rats [10]. We observed similar osteoprotective effects of cordycepin in both OVS and aged mouse models in our study, suggesting the potential of cordycepin for future clinical applications.

In conclusion, our study demonstrated the osteoprotective effects of cordycepin against H_2_O_2_ induced inhibition of osteogenesis by reducing oxidative stress both *in vitro* and *in vivo*. Cordycepin exerts this protective effects partially by increasing or decreasing expression of osteogenic and osteoclastogenesis marker genes as well as activating Wnt pathway activity. Cordycepin may represent a valuable regenerative medicine or a therapeutic reagent for treatment of age-related osteoporosis in humans.

## MATERIALS AND METHODS

### Isolation and culture of human BM-MSC

Bone marrow samples were obtained from 4 fetuses (median gestational age, 1315 weeks; range, 1112 to 1413). Single-cell suspensions of fetal bone marrow were prepared by flushing the bone marrow cells out of the humeri and femurs using a syringe and 22-gauge needle DMEM media supplemented with 10% fetal bovine serum (FBS) (Gibco, Grand Island, USA)penicillin (100 U/mL) (Sigma, St. Louisa, USA),, and streptomycin (100 μg/mL) (Sigma, St. Louisa, USA),. All cultured cells were incubated in a humidified atmosphere at 37°C and at 5% CO_2_. To expand the cells through successive passages, they were plated at 10^4^ cells/cm^2^, grown to near confluence, and harvested with the same protocol. To isolate individual colonies, nucleated cells were plated in a 100-mm dish at a density of 12 000/cm^2^ and were collected by cloning cylinders (Sigma, St. Louisa, USA) and expanded. Cultured fetal MSCs were recloned by single-cell sorting using FACS Vantage (Becton Dickinson, Franklin Lakes, USA).

### Drugs

Cordycepin with 98% purification was obtained following the extraction and separation using a column chromatographic method [31].

### Induction of osteoblast differentiation

Approximately 1×10^4^ cells BM-MSCs were placed in a 35 mm culture dish (Corning, Corning, USA). Osteoblast differentiation was induced by culturing cells in osteogenic medium composing of DMEM-high glucose, 10% FBS (Hyclone, Logan, USA), 100-100 μg/mL penicillin-streptomycin (Gibco, Grand Island, USA), 50 μg/mL L-ascorbic acid-2-phosphate (Sigma, St. Louisa, USA), L-glutamine (Sigma, St. Louisa, USA), 10-7 M dexamethasone (Sigma, St. Louisa, USA), and 10 mM β-glycerophosphate (Sigma, St. Louisa, USA) for 21 days. Media was changed every 3 days.

### Flow cytometry

BM-MSC cells were trypsinized and stained with anti-CD34-fluorescein isothiocyanate (FITC), CD45-FITC, CD73-FITC, CD90-FITC, CD105 (endoglin)-FITC, and were analyzed by FACScalibur flow cytometry (Becton Dickinson, Franklin Lakes, USA).

### Cell viability assay

For this experiment, the BM-MSC cells were treated with different concentration of H_2_O_2_ (0.1, 0.2, 0.5, 1 and 2 mM) for 24 h. Different concentrations of cordycepin (1, 5, 10, 20, 40 and 80 μg/mL) were supplemented in the culture medium during the whole culture. MTT assays were performed to measure cell survival. The absorbances of all of the wells were recorded using a micro-plate reader at 492 nm wavelength. The cell survival of the control group, which was not exposed to either H_2_O_2_ or cordycepin, was defined as 100%.

### Alkaline phosphatase (ALP) activity assay

BM-MSC cells (3 × 10^3^ cells/well) were incubated in a 96-well plate overnight and treated with different concentrations of H_2_O_2_ for 24h and/or the cordycepin in the medium for the whole duration of culture. ALP activity was measured in total cell lysates after homogenization in a buffer containing 1 mM Tris-HCl (pH 8.8), 0.5% Triton X-100, 10 mM Mg^2+^, and 5 mM p-nitrophenylphosphate as substrates. The absorbance was read at 405 nm. The ALP activity was normalized to total protein, which was measured using the Bradford protein assay. The assay was performed 8 days after cell culture.

### Calcium accumulation assay

To evaluate osteogenic differentiation, the calcium content in BM-MSCs was measured using Calcium Assay 16 days after cell culture (Genzyme Diagnostics, Charlottetown, PE, Canada) according to manufacturer's instructions. Briefly, samples were added with 1 M acetic acid and placed on a vortex overnight at 4°C to extract the calcium from the mineralized matrix. In a 96-well clear polycarbonate plate, 15 μL cell extract was mixed with 150 μL Calcium Assay reagent and incubated for 30 s at room temperature. The absorbance at 650 nm was determined using a SpectraMAX 250 microplate reader. The samples were measured in triplicate and compared to the calcium calibration curve. The calcium content was normalized by cell protein amount and expressed as relative calcium content normalized to control sample.

### Intracellular ROS levels determination

ROS level was measured using fluorescence associated oxidation of dichlorodihydrofluorescein (DCFH) to dichlorofluorescein (DCF; [32]). After treated with H_2_O_2_ and/or cordycepin, cells cultured on 8×8 mm square glass coverslips were rinsed with ice-cold phosphate-buffered saline (PBS) and then incubated in 10 μM DCFH-DA (Sigma, St. Louisa, USA) for 15 min at 37°C in the dark. Fluorescence was measured using confocal microscope (Zeiss LSM510) at excitation and emission wavelengths of 495 and 535 nm for DCF fluorescence. In average, ten BM-MSC cells/microscope field were quantified in three to four separate cultures per treatment condition. Zeiss confocal microscope was used to visualize DCF fluorescence. ImageJ was used to quantify fluorescence intensity.

### Real time-PCR

Total RNA was isolated from BM-MSCs treated with H_2_O_2_ and/or cordycepin, using TRIZOL reagent (Invitrogen, Carlsbad, USA). 1 μg RNA was initially reverse-transcribed into cDNA using the SuperScript™ III First-Strand Synthesis System (Invitrogen, Carlsbad, USA). Real-time quantitative PCR reactions were set up in triplicate with Ssofast Master Mix (Biorad) and run on a LightCycler® 480 (Roche, New Yok, USA). The genes *osteopontin (OPN)* and *collagen I* were measured to assess osteogenic differentiation. For osteoclast differentiation, *osteoprotegerin (OPG)* and *receptor activator of nuclear factor-κB ligand (RANKL)* were measured. The housekeeping gene, *glyceraldehyde-3-phosphate dehydrogenase (GAPDH)*, was used as an endogenous reference gene. Primers used in the experiment were as follows:

*OPN*, 5′-GAGACCCTTCCAAGTAAGTCCA (forward)

and 5′-GATGTCCTCGTCTGTAGCATCA (reverse);

*Collagen I*, 5′-ACAGCCGCTTCACCTACAGC (forward)

and 5′-TGCACTTTTGGTTTTTGGTCAT (reverse);

*OPG*, 5′-TGCTGTTCCTACAAAGTTTACG (forward)

and 5′-CTTTGAGTGCTTTAGTGCGTG (reverse);

*RANKL*, 5′-CCAGCATCAAAATCCCAAGT (forward)

and 5′-CCCCAAAGTATGTTGCATCCTG (reverse);

*Runx2*, 5′-TCTTAGAACAAATTCTGCCCTTT (forward)

and 5′-TGCTTTGGTCTTGAAATCACA (reverse);

*Axin2*, 5′-CTCCTTGGAGGCAAGAGC (forward)

and 5′-GGCCACGCAGCACCGCTG (reverse);

*c-MYC*, 5′-TGGATTTTTTTTCGGGTAGTGG (forward)

and 5′-GTCGTAGTCGAGGTCATAGTTCC (reverse);

*GAPDH*, 5′-GAAGGTGAAGGTCGGAGTC (forward)

and 5′-GAGATGGTGATGGGATTTC(reverse).

### Nuclear fraction and western blot

Cell lysate from the tissues was extracted using RIPA lysis buffer (Santa Cruz, Dallas, USA) containing 1 % protease inhibitor cocktail. For β-catenin analysis in the nucleus, cells were harvested and subjected to nuclear and cytoplasmic fractionation using NE-PER Nuclear and Cytoplasmic Extraction Reagents (Thermo Scientific, USA) according to the instruction. Briefly, cells were washed with PBS, centrifuged down and left dry. Reagents CER I and CER II were added followed by centrifugation. Supernatant that contains cytoplasmic extract was discarded. Reagent NER was added to dissolve the pallet, which contains nuclear extract. Extracts were subjected to further western blot analysis or stored at −80°C for further use. For electrophoresis, a total of 30 μg of protein were loaded onto a 12 % SDS-PAGE gel. After transfer, membranes were blocked in 5 % nonfat milk in Tris-buffered saline (TBS)/Tween-20 (0.2 %) overnight at 4°C and then incubated with rabbit polyclonal antibodies against β-catenin, cyclin-D1, H3 and GAPDH diluted in TBS/T for 1 h at37°C. After three washes in TBS/T, the membranes were incubated with anti-rabbit IgG conjugated to horseradish peroxidase (Zhongshan Golden Bridge Biotechnology, Beijing, China) at a dilution of 1:2,000 in TBS/T for 40 min at 37°C. The immunoreactive bands were visualized with an enhanced chemiluminescence kit (Zhongshan Golden Bridge Biotechnology, Beijing China).

### Animal experiments

Ovariectomized (OVX) mouse model was established as described before [33]. Briefly, forty eight 8-week-old, BALB/c female mice with similar weight were obtained from Shanghai Jiaotong University. After adaptation to the laboratory environment (a well-ventilated controlled room at 20°C on a 12-h light/dark cycle; the animals were given free access to water and food) for 1 week, the mice experienced sham-operation (*n* = 10) or were surgically ovariectomized (*n* = 40) under anesthesia using pentobarbital sodium (50 mg/kg body weight, i.p.). The ovariectomy operation was performed according to Steven K. Boyd's procedure [34]. For the aged animal model, eight young BALB/c female mice (8-week-old) and forty old BALB/c female mice (one-year-old) were obtained from Shanghai Jiaotong University and acclimatized to the facility for at least 1 week prior to the experiment. For *in vivo* osteogenesis experiments (Figure 7) in both mouse models, mice were divided into control group (*n* = 8) or cordycepin treated groups (4 groups, n=8 for each group). Experimental mice received i.p. injection of 1, 5, 10 and 20 mg/kg of cordycepin daily for four months. For the experiments in Figure 8, the animals received the different concentrations of cordycepin daily until the end of experiment (8 weeks). This study was carried out in strict accordance with the recommendations in the Guide for the Care and Use of Laboratory Animals of the National Institutes of Health (Guide for the Care and Use of Laboratory Animals, 9th edition). The protocol was approved by the Committee on the Ethics of Animal Experiments of Shanghai Jiao Tong University Affiliated Sixth People's Hospital. All surgery was performed under sodium pentobarbital anesthesia, and all efforts were made to minimize suffering.

### MDA, GSH measurement

The activity of malondialdehyde (MDA) in whole blood samples from OVX and aged mouse models receiving i.p. injections of 1, 5, 10 and 20 mg/kg of cordycepin was determined using a lipid peroxidation MDA assay kit according to the manufacturer's instructions. The binding of thiobarbituric acid to MDA results in a chromogenic complex that can be detected at 532 nm using a spectrophotometer. The activity of Glutathione (GSH) was determined using the GSH assay kit. The GSH activity was determined by the reaction of GSH with 5.50-dithiobis-2-nitrobenzoic acid (DTNB) to produce a product that could be measured using a spectrophotometer at 412 nm.

### *In vivo* osteogenesis of MSCs under cordycepin treatment

A total of 1 × 10^6^ MSCs was injected under the skin of the mice. After anesthesia with subcutaneous injection of a combination of 0.5 μL/g of tiletmine-zolazepam and 0.5 μL/g xylazine, small skin incisions were made and subcutaneous pouches were formed in the back of experimental animals, into which the MSCs were injected. After that, the skin incisions were closed with 4-0 nylons. For the cordycepin treated group, the mice were treated with cordycepin (1, 5, 10 and 20 mg/kg) during the whole experiment. To measure the calcium contents of *in vivo* implanted specimens, each sample was deparaffinized, dried at 95°C for 1 h, weighed and decalcified in 1 mL of Calci-clear Rapid. The calcium content of the supernatants was determined by spectrophotometry using Methylxylene blue method.

### Hematoxylin-eosin (H&E) staining

The femurs were cleaned and placed in decalcifying solution and 10% formic acid in PBS for 24 h, then dehydreated in 95% ethanol and embedded in paraffin. The sections were cut and stained with H&E and examined by light microscopy.

### Micro-computed tomography (micro-CT) examination

The femurs were evaluated by micro-CT (Scanco Medical AG, Switzerland). The scanning parameters were set at 50 kV, 100 μA. Images were reconstructed and analyzed with Scanco Medical software. Fractional bone volume is determined as bone volume per tissue volume, while bone mineral density was calculated.

### Statistical analysis

Results are expressed as mean±SEM, and statistical comparisons were performed using the Student t test. Statistical difference was considered to be significant only if *P* <0.05.

## SUPPLEMENTARY MATERIAL FIGURE


