# Supplementary material for: Cordycepin prevents oxidative stress-induced inhibition of osteogenesis
Source: Oncotarget. 2015 Oct 10;6(34):35496–508. doi: 10.18632/oncotarget.6072 (PMC4742120; doi:10.18632/oncotarget.6072)
Supplement: Supplementary file 1 [file oncotarget-06-35496-s001.pdf]

Cordycepin prevents oxidative stress-induced inhibition of osteogenesis

Supplementary Material

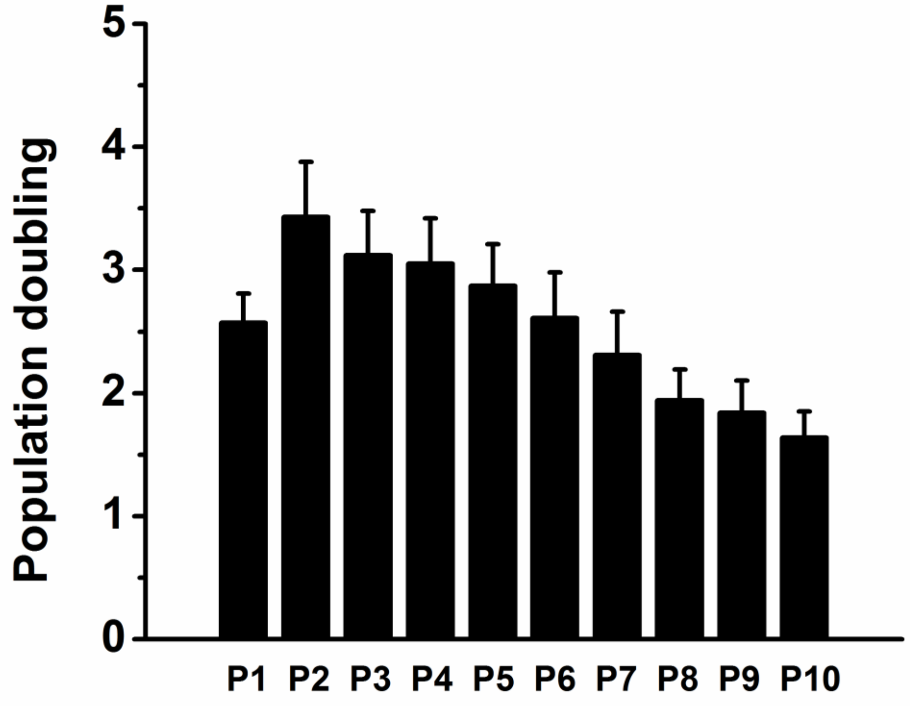

Figure S1. Growth kinetics of BM-MSCs. The population-doubling (PD) in passages 1-10; The PD time in P5-P6 was determined to be ~64 hours.
